# Assembly of Polyacrylamide-Sodium Alginate-Based Organic-Inorganic Hydrogel with Mechanical and Adsorption Properties

**DOI:** 10.3390/polym11081239

**Published:** 2019-07-26

**Authors:** Yiying Yue, Xianhui Wang, Qinglin Wu, Jingquan Han, Jianchun Jiang

**Affiliations:** 1 College of Biology and the Environment, Nanjing Forestry University, Nanjing 210037, China; 2 School of Renewable Natural Resources, Louisiana State University Agricultural Center, Baton Rouge, LA 70803, USA; 3 College of Materials Science and Engineering, Nanjing Forestry University, Nanjing 210037, China; 4 College of Chemical Engineering, Nanjing Forestry University, Nanjing 210037, China; 5 Institute of Chemical Industry of Forest Products, Chinese Academy of Forestry, Nanjing 210042, China

**Keywords:** hydrogel, inorganic nanofillers, interpenetrating polymer network, mechanical behavior, adsorption properties

## Abstract

Hydrogels have been widely used in water purification. However, there is not much discussion and comparison about the effects of different nanofillers on the reinforcement and adsorption performances of hydrogels, which can be subjected to rapid water flow and possess strong adsorption ability. In this work, polyacrylamide (PAAM)-sodium alginate (SA) interpenetrating polymer network-structured hydrogels were prepared by in situ polymerization. PAAM formed the first flexible network and SA constructed the second rigid network. Three kinds of inorganic nanoparticles including carbon nanotubes (CNTs), nanoclays (NCs), and nanosilicas (NSs) were incorporated into a PAAM-SA matrix via hydrogen bond. The obtained hydrogels exhibited a macroporous structure with low density (≈1.4 g/cm^3^) and high water content (≈83%). Compared with neat PAAM-SA, the hydrogels with inorganic nanoparticles possessed excellent mechanical strengths and elasticities, and the compression strength of PAAM-SA-NS reached up to 1.3 MPa at ε = 60% by adding only 0.036 g NS in a 30 g polymer matrix. However, CNT was the best filler to improve the adsorption capacity owing to its multi-walled hollow nanostructure, and the adsorption capacity of PAAM-SA-CNT was 1.28 times higher than that of PAAM-SA. The prepared hydrogels can be potential candidates for use as absorbents to treat wastewater.

## 1. Introduction

Currently, rapid industrialization has created a multitude of pollutants to the environment [1,2,3,4]. Among the hazardous wastes, heavy metal pollution has become one of the most serious problems [2]. Heavy metal ions are difficult to degrade into less toxic or non-toxic compounds, and they threaten the safe use of water resources [2,5]. Among many metal ions, copper ion (Cu^2+^) represents one of the most important problems due to inappropriate discharges in the smelting and mining industries [5]. Excessive intake of Cu^2+^ induces serious neurodegenerative diseases and causes damage to living organisms [1]. Many treatment techniques have been adopted for removing Cu^2+^ from aqueous streams, and adsorption is considered to be a promising technology because of its easy operation, high efficiency, wide application range and good cost effectiveness [2,5,6]. However, the addition of nanoadsorbents with a high specific surface area and a high reactivity to water requires a subsequent separation process, such as centrifugation and filtration [2,6]. Thus, these adsorbents cannot apply to commercial levels. Hydrogels, three-dimensional hydrophilic materials capable of holding large amounts of water, have been widely used in water purification because of their easily reusable, biodegradable, and environmentally benign properties [7,8]. Nevertheless, most of the hydrogels suffer from a lack of mechanical performance. The low mechanical strength and poor rheological performance cannot withstand the high levels of stress and strain needed for water flow, which severely limit their further practical application in wastewater treatments [2,9]. Therefore, during the past decade, tremendous efforts have been devoted to the development of high-strength hydrogels.

Constructing an interpenetrating polymer network (IPN) that is based on two independently cross-linked polymer networks is an effective method to enhance the stiffness and strength of hydrogels [5,10]. With enhanced mechanic strength and better rheological performance, hydrogels could be easily separated from an aqueous environment. An IPN-structured hydrogel could be constructed by chemically or physically cross-linking polyacrylamide (PAAM) and sodium alginate (SA) [10,11]. PAAM with the side amine and carbonyl functional groups could effectively adsorb heavy metal ions [12]. SA is one of the natural polysaccharides consisting of mannuronic acid (M) and guluronic acid (G) groups. G sequences in SA can be complex with Ca^2+^ to form “egg box” junctions, while PAAM can be easily cross-linked with *N*,*N*′-methylenebis-acrylamide (MBA) by covalent bonding [2,8]. The obtained PAAM-SA hydrogel benefits from a mixing covalent and the ionic cross-linking is highly stretchable and tough. 

The mechanical and adsorptive performance of IPN-structured hydrogels can be further improved by incorporating nanoparticles [8,13]. The reinforcing fillers, such as multi-walled carbon nanotubes (CNTs) [14], nanoclays (NCs) [15] and nanosilicas (NSs) [16] have been introduced into the hydrogel matrix. Owing to their low mass density, high mechanical strength, large specific surface area, and superior compatibility with the polymer matrix, CNTs, NCs and NSs are expected to effectively reduce the brittleness and improve the sorption capacities of hydrogel [17,18,19]. Chatterjee et al. verified that the adsorption capacity of chitosan beads was greatly improved by introducing CNTs [14]. Nguyen et al. proved that the heavy metal removal capacity was highly related to the content of NC in chitin hydrogel [7]. In addition, the presence of NS enhancing the porosity of the hydrogel network and further affecting the adsorption capacity of chitosan-g-acrylic acid hydrogel has been reported by Pourjavadi et al. [20]. However, there is not much discussion and comparison about the effects of different nanofillers on the reinforcement and adsorption performances of hydrogels, which have good mechanical strength and a strong adsorption ability and can be used in alleviating the severe environmental situation. 

Based on this consideration, a series of hydrogels were fabricated by incorporating three different reinforcing fillers in a cross-linking network. SA and PAAM acted as the polymer matrix, providing rigidity and ductility through ionic and covalent cross-linking. Three kinds of nanofillers—CNT, NC and NS—were introduced into the PAAM-SA matrix to form ternary composite hydrogels. The effects of CNT, NC, and NS on the density and water content of the hydrogel were investigated. Compressive and rheological tests were performed to study the mechanical behavior of the hydrogels. A batch of experiments was devoted to examining the adsorption capacity of the hydrogels. A formation mechanism was also proposed for a thorough understanding of the interaction between the hydrogel matrix and the inorganic nanofillers. This novel organic-inorganic nanocomposite material is a good candidate for alleviating the problem of water pollution. 

## 2. Materials and Methods

### 2.1. Materials

Acrylamide (AM), sodium alginate (SA, mannuronate/guluronate ratio of the alginate = 1:1, MW = 140,000) [21], *N*,*N*′-methylenebis-acrylamide (MBA), potassium persulphate (KPS), CuCl_2_, and CaCl_2_ were purchased from Aladdin Industrial Co. (Shanghai, China). Nanoclays (NCs) and nanosilicas (NSs) were purchased from Deke Island Gold Co. (Beijing, China). Aligned multi-walled carbon nanotubes (CNTs) were purchased from Zhongke Timesnano (Chengdu, China). All reagents and solvents were of analytical grade. Deionized water was used for all the experiments.

### 2.2. Synthesis of Hybrid Hydrogels

**Figure 1:**
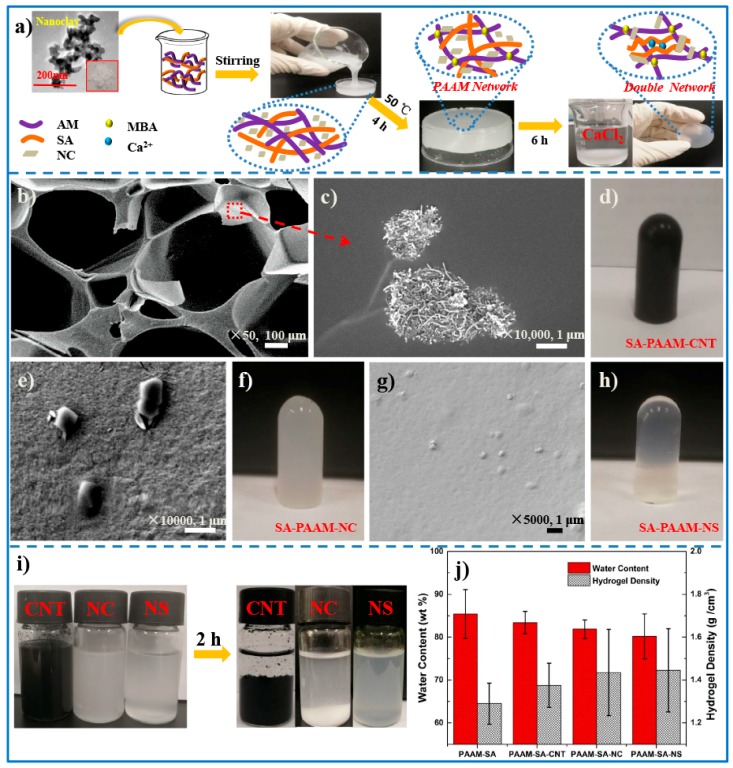
The morphology of hydrogels: (**a**) schematic illustration of the hydrogel fabrication; (**b**) SEM photographs of freeze-dried interpenetrating polymer network (IPN)-structured hydrogels; amplified view of (**c**) carbon nanotubes (CNTs), (**e**) nanoclays (NCs), and (**g**) nanosilicas (NSs) distributed in polymer matrix; photographs of (**d**) PAAM-SA-CNT, (**f**) PAAM-SA-NC and (**h**) PAAM-SA-NS; (**i**) appearance of CNT, NC, NS suspensions after 2 h of settling; (**j**) water content and density of hydrogels.

A mass of 0.036 g CNT was first dispersed in 25 mL distilled water, then amounts of 3.6 g AM and 0.60 g SA were dissolved in the above CNT aqueous dispersion at 50 °C until a homogenous suspension was formed. Subsequently the cross-linking agent MBA (0.018 g) and the initiator KPS (0.036 g) were introduced and the mixture was stirred vigorously for 1 h. Following degassing, the mixture was injected into a reaction mold at 50 °C for 4 h to build the first covalent network. Afterwards, the as-prepared hydrogel was transferred to a 0.5 mol/L CaCl_2_ solution and kept for 6 h to construct the IPN-structured hydrogel [10]. The obtained hydrogel was designated as PAAM-SA-CNT. The preparation of PAAM-SA-NC and PAAM-SA-NS was exactly the same as the fabrication of PAAM-SA-CNT, except for replacing 0.036 g CNT with 0.036 g NC and 0.036 g NS, respectively. Due to the high viscosity of sodium alginate, inorganic nanofillers can be well dispersed in the aqueous SA solution. To investigate the efforts of inorganic nanoparticles on hydrogels, PAAM-SA was also prepared by introducing 0.60 g SA in 25 mL distilled water. The schematic illustration of the hydrogel fabrication is displayed in Figure 1. 

### 2.3. Characterizations

#### 2.3.1. Morphology of Inorganic Nanoparticle Dispersions and Prepared Hydrogels

A transmission electron microscope (JEM-1400, JEOL, Tokyo, Japan) with an accelerating voltage of 80 kV was employed to observe the morphology of the CNT, NC and NS aqueous suspensions. The dimensions of the captured CNT, NS, and NC were analyzed using Image J software (version v18.0, U.S. National Institutes of Health, Bethesda, MD, USA). For each sample, the lengths of the inorganic nanoparticles were recorded based on over 10 randomly selected TEM images. The morphology of the freeze-dried hydrogel samples was analyzed using field emission scanning electron microscopy (JSM-7600F, JEOL, Tokyo, Japan) at an accelerating voltage of 5.0 kV. 

#### 2.3.2. Density and Water Content of Hydrogels 

The hydrogel with a certain mass (*M*) was placed in a graduated cylinder containing a certain volume of water (*V*_0_). The water volume was changed to *V*_1_ after introducing the hydrogel to the container. The density (ρ) of the hydrogels was calculated using Equation (1):

(1) $$\rho =\frac{M}{V_{1}-V_{0}}$$

To determine the water content of the hydrogel, each sample with an initial weight of *W_i_* was dried in a vacuum oven (DHG-9023A, Jinghong Instrument co., Shanghai, China) at 50 °C until a constant weight of *W_d_*. The water contents (*W_c_*) of the hydrogels were calculated using Equation (2):

(2) $$W_{c}=\frac{W_{i}-W_{d}}{W_{i}}\times 100\%$$

Three repeated measurements were conducted for the densities and water contents of the hydrogels.

#### 2.3.3. Formation of 3D Network and Structural Analysis of NC

An X-ray photoelectronic spectroscopy (XPS) machine equipped with a high-resolution spectrometer (AXIS UltraDLD, Shimadzu, Tokyo, Japan) was employed to analyze the structure and constituent of the NC. The binding energy was corrected using the C1s of 284.8 eV. Fourier-transform infrared spectrometry (FTIR) spectra were acquired on a VERTEX 80v spectrometer (Thermo Fisher Scientific Inc., Waltham, MA, USA) in the spectral range of 4000–400 cm^−1^ with a resolution of 4 cm^−1^. 

#### 2.3.4. Compressive and Rheological Measurements

Uniaxial compressive strength measurements were carried out on cylindrical samples (thickness of 20 mm; diameter of 38 mm) using a mechanical testing instrument (TY-8000B, Tianyuan machinery Co., Jiangsu, China). The compression was conducted at a constant rate of 5 mm/min and the compression force was applied to the samples with the maximum loading of 5 kN. Rheological properties were tested using a HAAKE RheoStress 600 Rheometer (Thermo Fisher Science Inc. Waltham, MA, USA) equipped with 35 mm diameter plate-and-plate geometry at 25 °C. The dynamic strain sweep was determined from 0.1 to 100% at a frequency of 1.0 Hz, and the frequency sweep was carried out to record storage modulus and loss modulus at different frequencies of 0.1 to 100 rad/s. 

#### 2.3.5. Adsorption/desorption of Metal Ion (Cu^2+^)

Adsorption experiments were carried out by using 120 mg prepared hydrogels in 150 mL of Cu^2+^ solution at 25 °C. Various Cu^2+^ concentrations (20–160 mg/L) were applied to the experiment. After reaching absorbent equilibrium, the adsorbents were removed from the solution. The concentrations of Cu^2+^ were determined using a flame atomic absorption spectrometer (FAAS, TAS-990, Persee, Beijing, China). Adsorption capacity at equilibrium *q_e_*(mg/g) was calculated with the following equation:

(3) $$q_{e}=\left(C_{0}-C_{e}\right)\times \frac{V}{m}$$

where *C*_0_ (mg/L) is the initial concentration and *C_e_* (mg/L) is the equilibrium concentrations of Cu^2+^, *V*(*L*) is the volume of the Cu^2+^ solution, and *m*(*g*) is the mass of the sample.

The reusability of four types of hydrogels was examined through four adsorption–desorption cycles after regeneration by distilled water at room temperature for 10 min. 

## 3. Results and Discussion

### 3.1. Morphology of IPN-Structured Hydrogels and Inorganic Nanoparticles

As shown in Figure 1a, the inorganic nanofiller-reinforced IPN-structured hydrogels were successfully fabricated. To better understand the network architecture of the designed hydrogels, the materials were imaged by SEM. Figure 1b shows that PAAM-SA-CNT possessed an interconnected macroporous structure. Because of the high water content of hydrogels (≥80%, Figure 1j), the diameters of pores in hydrogels were in the range of 300 to 800 μm. This porous structure contributed greatly to the high adsorption capacity of the hydrogels. Due to the low loading level of inorganic nanofillers, the macroporous structure of PAAM-SA-NC and PAAM-SA-NS was similar to that of PAAM-SA-CNT. Therefore, the morphology of PAAM-SA-NC and PAAM-SA-NS was not displayed. The morphologies of CNT, NC, and NS distributed in the polymer matrix were observed at a high magnification. Compared with CNT and NC, which were agglomerated to micron scale (Figure 1c,e), NS exhibited a better dispersion (Figure 1g). The appearances of CNT, NC, and NS dispersions shown in Figure 1i further confirmed this conclusion. The images of PAAM-SA-CNT, PAAM-SA-NC, and PAAM-SA-NS are displayed in Figure 1d,f,h, respectively. The high homogeneity of all the prepared hybrid hydrogels suggested that these inorganic nanofillers were uniformly dispersed in the hydrogel matrix, leading to the increase in cross-linking densities (Figure 1j). These results were in agreement with previously published information [22]. 

**Figure 2:**
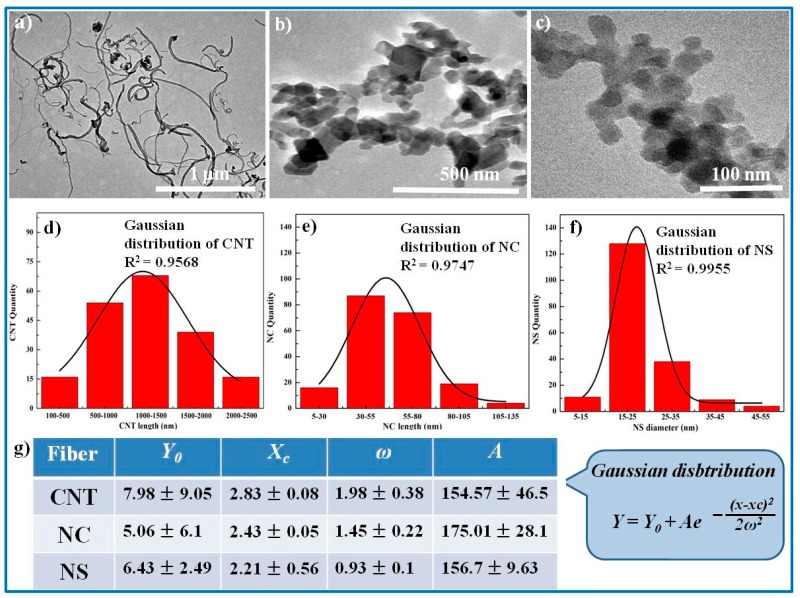
The morphology of inorganic nanoparticles: TEM pictures of (**a**) CNT, (**b**) NS, and (**c**) NC, respectively; the dimension histogram of (**d**) CNT, (**e**) NS, and (**f**) NC, respectively; and (**g**) the statistical parameters in Gaussian distribution for inorganic nanoparticles.

The micrographs of CNT, NC, and NS are presented in Figure 2a–c. As shown in Figure 2a, CNT exhibited a long wire-like shape with an average length of 1260 ± 644 nm and aspect ratio of 79.2, whereas NC was flaky with an average length of 58 ± 16 nm (Figure 2b) and NS was spheroidal with an average diameter of 23 ± 6.4 nm (Figure 2c). The size distributions of CNT, NS and NC were statistically analyzed based on the TEM images, and the results are displayed in Figure 2d–f, respectively. Their length was consistent with Gaussian distribution (Figure 2g). It can be seen that NS possessed the smallest size among these three particles. Thus, NS exhibited favorable dispersibility and superiority in distributing on the PAAM-SA matrix. In contrast, the larger length of CNT and NC led to easier agglomeration and deposition (Figure 1j). These conclusions were in accordance with the results observed in the SEM images.

### 3.2. Formation Mechanism for Hybrid Hydrogels

**Figure 3:**
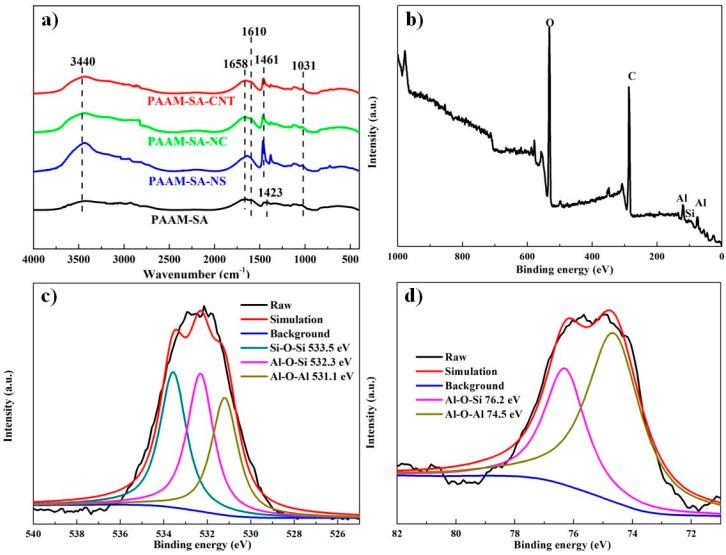
(**a**) FTIR spectra of PAAM-SA, PAAM-SA-CNT, PAAM-SA-NC, PAAM-SA-NS; (**b**) XPS spectrum of nanoclay; XPS spectra of curve-fitted (**c**) O1s and (**d**) Al2p peaks.

**Figure 4:**
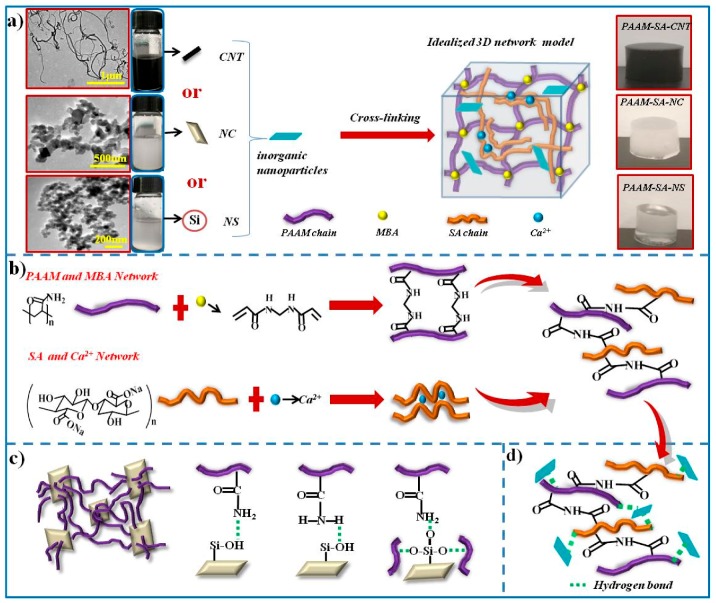
Schematic illustration of (**a**) the 3D network of the PAAM-SA-based hydrogels; (**b**) the formation of the PAAM and MBA network, the SA and Ca^2+^ network, and the interaction between the PAAM and SA networks; (**c**) the formation of hydrogen bonding in PAAM-SA-NC; and (**d**) the intermolecular bonding between the PAAM-SA matrix and the inorganic nanofillers.

Figure 3a shows the infrared spectra for PAAM-SA, PAAM-SA-CNT, PAAM-SA-NC, and PAAM-SA-NS. The gelation mechanism of the hydrogel is presented in Figure 4a. For the IR spectra, it was clear that the hybrid hydrogel showed a broad peak at 3440 cm^−1^ for the OH group. The Na–O vibration in SA appeared at 813 cm^−1^ (Figure S1, Supplementary Materials) was not observed in the spectra of all the hybrid hydrogels, and the carboxyl group peak in SA was shifted from 1413 to 1423 cm^−1^, indicating the cross-linking of SA with Ca^2+^ (Figure 4b) [23]. The characteristic absorption peaks of PAAM at 1610 cm^−1^ for N–H can be found in the spectra of all the hydrogels, implying the presence of PAAM. The absorption peaks at 1658 and 1461 cm^−1^ suggested the development of new hydrogen bonds between the COO- groups on SA and the CONH_2_ groups on PAAM [24], which evidenced the formation of the IPN structure. After incorporating inorganic nanofillers, the intensity of hydroxyl vibration at 3440 cm^−1^ of the hydrogels increased, implying that intermolecular hydrogen bonds between the nanofillers and the PAAM-SA matrix were formed. The intensities of the peaks observed at 470 cm^−1^ (Si–O out-of-plane deformation), 1110 cm^−1^ (Si–O–Si stretching), and 966 cm^−1^ (Si–OH stretching) in the spectrum of the NS (Figure S1, Supplementary Materials) were greatly reduced in the spectra of the PAAM-SA-NS hydrogel, indicating the formation of hydrogen bonding between NS and the PAAM-SA matrix [25,26,27]. Furthermore, it can be seen that the peak intensities at 1461 cm^−1^ in PAAM-SA-CNT, PAAM-SA-NC, and PAAM-SA-NS were larger than that in PAAM-SA, which was also evidence of the development of possible interactions. A similar observation has been reported previously [28].

The schematic representation of the formation of intermolecular hydrogen bonding in PAAM-SA-based hydrogels is shown in Figure 4c. As shown in Figure 3b–d, XPS spectrum of the nanoclay confirmed the presence of Al2p (≈75 eV), Al2s (≈120 eV), and Si2p (≈104 eV) [29]. The sample showed the C1 peak at approximately 286.6ev, which is usually attributed to the free contamination [30,31]. After Gaussian cure fitting, three components were displayed to form the O1s photoelectron peak, which were associated with Si–O–Si, Si–O–Al and Al–O–Al bonds [32]. The Si–O–Al and Al–O–Al curves could be further evidenced by the deconvolution of the Al2p photoelectron peak.

### 3.3. Mechanical Properties 

**Figure 5:**
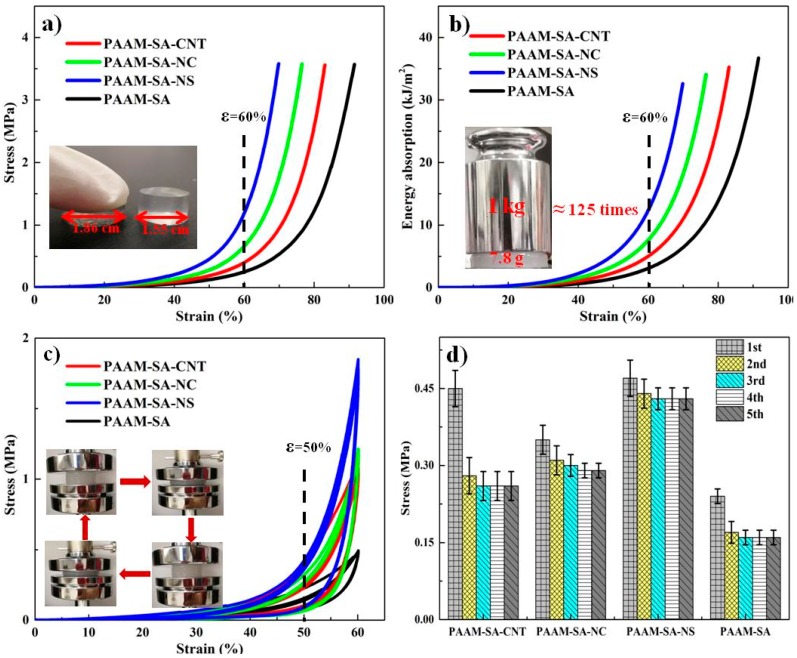
Compression stress–strain behavior of hydrogels: (**a**) compression plots; (**b**) energy absorption curves; (**c**) hysteresis of hydrogels for five cycles; and (**d**) stresses at 50% strain under five successive loadings.

To examine the reinforcement efforts of the nanofillers, a series of compression measurements were performed on PAAM-SA, PAAM-SA-CNT, PAAM-SA-NS, and PAAM-SA-NC. The stress–strain curves of IPN-structured hydrogels are presented in Figure 5a. At the initial stage (<40% initial strain), the stress exhibited a slowly increasing trend. As the strain increased, the stress increased sharply, implying the hydrogels exhibited elastic deformation and stored energy to resist the applied load [33]. The compression stress and specific compressive stress (in consideration of actual density) of PAAM-SA were 0.27 ± 0.1 MPa and ≈0.2 MPa·cm^3^/g at ε = 60% (Table 1). After incorporating inorganic nanofillers, all the hydrogels sustained higher stress, and the compressive strength was in the order of PAAM-SA-NS > PAAM-SA-NC > PAAM-SA-CNT > PAAM-SA, implying the inorganic nanofillers played a crucial role in improving mechanical strength. The integration of NS significantly enhanced the mechanical properties of the PAAM-SA matrix (Table 1). This phenomenon indicated that homogeneously distributed NS had an excellent reinforcing effect on the composite hydrogel. The possible reason was that the well-dispersed NS could interact with PAAM and SA through hydrogen bonds, resulting in the prevention of micro-cracks. Compared with NS, the reinforcing efforts of CNT and NC on the hydrogel matrix were not significant due to their larger dimension and tendency of aggregation. 

The energy absorption–strain curves of all the prepared hydrogels are shown in Figure 5b. The energy absorption plots followed the trend of PAAM-SA-NS > PAAM-SA-NC > PAAM-SA-CNT > PAAM-SA. PAAM-SA-NS reached a value of 13.5 kJ/m^3^ at ε = 60%, which was approximately 4-fold larger than that of PAAM-SA (Table 1). The photograph inserted in Figure 5b shows that PAAM-SA-NS with a water content of 80.2% can support more than ≈125 times its own weight without any distortion. 

Because an excellent fatigue resistance and a rapid self-recovery property are critical parameters for load-bearing applications of hydrogels [34], the hydrogels were subjected to five cyclical compression tests and the resulting data are shown in Figure 5c. The stress increased slightly at initial strain (<50%) followed by a rapid rise at 50–60% strain. After removing the compression force, a pronounced hysteresis was exhibited, indicating that the energy dissipated in the polymer matrix [18]. All subsequent cycles followed the same procedure as the first one, and there was no time interval between any two cycles. As shown in Figure 5c, during these five loading–unloading cycles, the hydrogels were compressed to previously ultimate deformation and the strains could return to 0% after removing the force, suggesting an effective recovery and negligibly permanent deformation in the recycling [35]. Additionally, after an obvious hysteresis in the first cycle, a significant overlapping of the cyclic compression curves was observed. The possible reason was that the interstitial water located in the hydrogel was squeezed out during the first loading and that water could not come back before the subsequent compression cycles, impeding the re-swelling to the initial state [36].

Figure 5d shows the stresses at 50% strain under five cyclic loadings. Compared with other hydrogels, PAAM-SA-NS had a slight change in hysteresis among the compression cycles, which may be because the smaller size and larger specific area of NS made it more liable to homogenously disperse and easily form hydrogen bonds between itself and the matrixes. As the strain increased, PAAM-SA-NS with more hydrogen bonding could resist larger external loading. As a result, the resilience and anti-fatigue performances of PAAM-SA-NS were enhanced. The results of five cyclic compression tests demonstrated that all the prepared hydrogels displayed excellent recoverability. Therefore, the hydrogels can be promising candidates for load-bearing applications. 

### 3.4. Rheological Characterization

**Figure 6:**
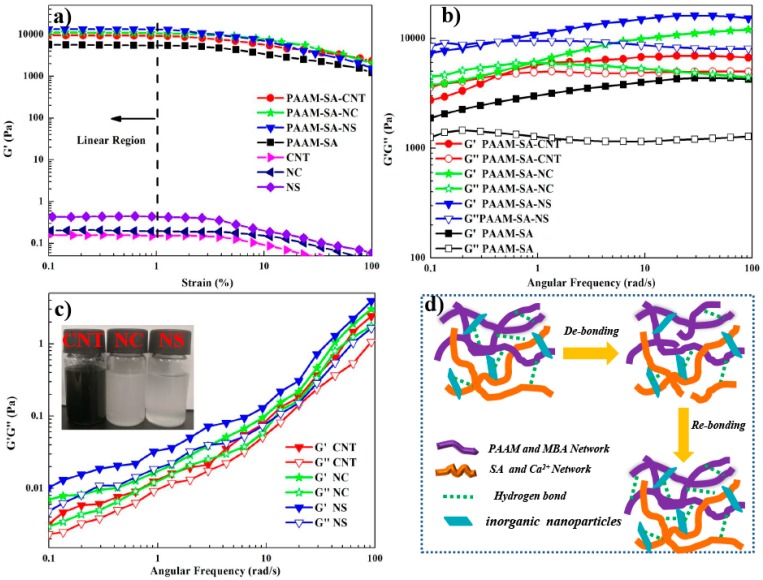
Dynamic viscoelasticity performance of the hydrogels at 25 °C: (**a**) strain dependence of storage modulus of hybrid hydrogels and 0.1% inorganic nanoparticle suspension at frequency of 1.0 Hz; (**b**) frequency dependence of storage and loss modulus of hydrogels; (**c**) frequency dependence of storage and loss modulus of 0.1% inorganic nanoparticle suspension; and (**d**) schematic illustration of the mechanism of fracture and rebuilding of hydrogen bonds.

Various dynamic oscillatory rheological measurements were performed on the hydrogels and 0.1 wt % inorganic nanoparticle suspensions to further investigate the ductility and viscoelasticity of the hydrogel. The linear viscoelastic region (LVR) was determined by strain sweep test, where the storage modulus (*G*′) and the loss modulus (*G*′′) were independent to the applied strain. As shown in Figure 6a, the hydrogel presented a relatively high *G*′*_max_*, whereas *G*′*_max_* for the inorganic nanoparticle suspension were comparatively low, suggesting the toughness of the hydrogel. Compared with PAAM-SA, the hydrogels with inorganic nanoparticles presented a relatively higher *G’_max_*, and the *G*′*_max_* of the hydrogels followed the order of PAAM-SA-NS > PAAM-SA-NC > PAAM-SA-CNT > PAAM-SA. For all the hydrogels, when strain (*γ*) was lower than 1.0%, the *G*′ was relatively constant. Therefore, a strain level of 1.0% was chosen to study the viscoelastic behavior of each hydrogel. 

Dynamic frequency sweep tests were further performed for the hydrogels and inorganic nanoparticle suspensions, and the *G*′ and *G*′′ values of the hydrogels are displayed in Figure 6b,c, respectively. It can be seen that the *G*′ and *G*′′ of the hydrogels had a similar trend. All the hydrogels had higher *G*′′ than *G*′ at low frequencies, implying a typical elastic behavior. As *ω* increased, *G*′′ was gradually getting closer to *G*′ until an intersection of *G*′ and *G*′′ was achieved. This observation suggested that multiple reversible interactions were formed [37]. After the crossover point, *G*′ was higher than *G*′′, indicating a predominantly solid-like property [38]. For PAAM-SA, *G*′ and *G*′′ values at *ω* of 100 rad/s were 4275 and 1186 Pa, respectively. The high viscoelastic parameters confirmed the formation of the IPN structure. The values of *G*′ and *G*′′ significantly increased after incorporating inorganic nanoparticles (Figure 6b). Compared with the other hydrogels, PAAM-SA-NS exhibited the highest *G*′ (16066 Pa) and *G*′′ (8069 Pa) at *ω* of 100 rad/s. The possible reason was that NS with a smaller particle size could induce more cross-linking with the polymer matrix. As shown in Figure 6d, the hydrogen bonds were partially destroyed by the increasing frequency; in the meantime, a part of the broken hydrogen bonds could be reformed and entanglements among polymer chains could be reconstructed. 

For inorganic nanofiller suspensions, *G*′ was higher than *G*′′ throughout the whole *ω* range and both *G*′ and *G*′′ increased as ω increased (Figure 6c), indicating the original equilibrium network was partially disturbed and the nanoparticles had the ability to rearrange and construct an ordered network. Among all the nanoparticles, the *G*′ and *G*′′ of NS were approximately 1.7 and 1.57 times higher than those of CNT, confirming the significant enhancement effect of NS.

### 3.5. Adsorption Performances

**Figure 7:**
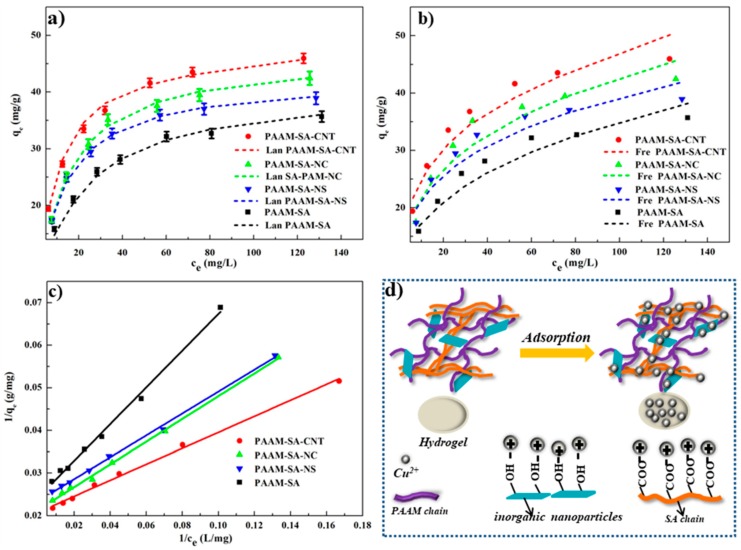
The adsorption capacity of hydrogels: (**a**) Langmuir adsorption isotherms and experimental results of Cu^2+^ on hydrogels; (**b**) Freundlich adsorption isotherms and experimental results of Cu^2+^ on hydrogels; (**c**) linearized Langmuir isotherms determined from 1/q_e_ versus 1/c_e_; and (**d**) mechanism of Cu^2+^ adsorption on hydrogels.

The adsorption performance of Cu^2+^ on the IPN-structured hydrogels was studied at 25 °C. The adsorption capacities of the hydrogels to Cu^2+^ at pH ranging from 2 to 6 is shown in Figure S2, Supplementary Materials. The experimental results at the pH level of 6 are shown in Figure 7. At a low initial concentration (≤40 mg/L), due to the presence of a large number of active groups and the exposing of complementary cavities on the surface of the hydrogel, a rapid increase in the number of adsorbed Cu^2+^ was observed. In addition, in the initial stage, the difference in concentrations between the hydrogel and Cu^2+^ also contributed to the increasing rate of adsorption [39,40]. As Cu^2+^ concentration increased, the adsorption rate became slower and the mass of Cu^2+^ on the hydrogels progressively increased. The possible reason was that the active sites on the surface of the hydrogel were gradually occupied owing to the limited adsorption activity of the hydrogel [41]. Subsequently, the adsorption rate continued to decline until a plateau was reached. The adsorption capacities for all the hydrogels were greater than 35.7 mg/g at 25 °C, which was better than most of the other adsorbents previously reported in the literature (Table 2). There were many factors influencing the metal ion adsorption, such as the chemical properties of adsorbent surface, the specific surface area of the adsorbent, and the interaction between Cu^2+^ and the hydrogel [12,42]. The porous nature of the hydrogel, a large quantity of carboxyl groups on SA, and the structural stability of the IPN structure resulted in an excellent adsorption capacity of the hydrogel. Another important reason for achieving the high adsorption capacity was that the formation of a strong coordination bond between Cu^2+^ and the electron-rich groups, such as a hydroxyl or a carboxyl group, led to a high affinity [43]. With the incorporation of the nanoparticles, the adsorption capacity of the hydrogels increased (Figure 7). The possible reason was that the active groups on the inorganic nanoparticles generated more adsorption sites, leading to a synergistic effect of the inorganic nanoparticles and the PAAM-SA matrix on adsorption. The adsorption mechanism is shown in Figure 7d. The adsorption capabilities were in the order of PAAM-SA-CNT > PAAM-SA-NC > PAAM-SA-NS > PAAM-SA. PAAM-SA-CNT possessed a better adsorption performance than PAAM-SA-NC and PAAM-SA-NS because CNT, with a high specific surface area (multi-walled hollow structure), could induce more free energy for adsorbing [17].

The Langmuir and Freundlich isotherms are the two most commonly used models to analyze the adsorption processes. The Langmuir isotherm model is suitable for describing a monolayer adsorption occurring on a finite number of homogeneous sites [48,49,50], whereas the Freundlich isotherm model is applicable for multi-molecular adsorption on a heterogeneous surface [51]. Langmuir and Freundlich isotherm models were used to determine the adsorption process, and the equations of these two models are described as Equations (4) and (5), respectively:

(4) $$q_{e}=(q_{m}K_{L}C_{e})/\left(1+K_{L}C_{e}\right)$$

where *q_m_* is the maximum adsorption capacity (mg/g), *C_e_* is the equilibrium concentration of Cu^2+^ in the aqueous solution (mg/L), and *K_L_* is a Langmuir adsorption equilibrium constant that reflects the adsorption performance of the adsorbent (L/mg);

(5) $$q_{e}=K_{F}C_{e}^{1/n}\;$$

where *K_F_* is the adsorption capacity for the Langmuir isotherm (mg/g), and *n* is related to adsorption intensity. 

Both Langmuir and Freundlich isotherms can be expressed in linearized form and are presented as Equations (6) and (7), respectively. The values of the isotherm parameters (*K_L_*, *q_m_*, *K_F_*, *1*/*n*) can be calculated by slope and intercept. The evaluated constants and the correlation coefficients (*R*^2^) of these two isotherm models are presented in Table 3:

(6) $$1/q_{e}=\left(1/K_{L}q_{m}\right)\left(1/C_{e}\right)+1/q_{m}$$

(7) $$logq_{e}=\frac{1}{n}logC_{e}+logK_{F}$$

As shown in Figure 7c and Table 3, the results of linear fitting indicated that the experimental data obeyed the Langmuir isotherm model well (*R*^2^ ≥ 0.99), which revealed that the adsorption of Cu^2+^ on the hydrogel was mainly monolayer. Furthermore, the values of the calculated maximum adsorption of Cu^2+^ (*q_m_*) for all the hydrogels were very close to the experimental values, further indicating that the surface structure of the hydrogel was homogeneous and that monolayer adsorption had occurred. With the addition of inorganic nanoparticles, the *K_L_* values of the hybrid hydrogels were higher than that of PAAM-SA, suggesting that the inorganic nanoparticles had a higher affinity to Cu^2+^. 

*R_L_* indicates the favorability or unfavorability of the Langmuir isotherm, which can be either irreversible (*R_L_* = 0), favorable (0 < *R_L_* < 1), linear (*R_L_* = 1), or unfavorable (*R_L_* > 1) [52]. The expression of *R_L_* is shown in Equation (8):

(8) $$R_{L}=1/\left(1+C_{0}K_{L}\right)$$

The constants *R_L_* for all hydrogels were less than one (Table 3), which suggested a satisfactory affinity between the hydrogels and Cu^2+^ [53]. 

### 3.6. Reusability of Hydrogels

**Figure 8:**
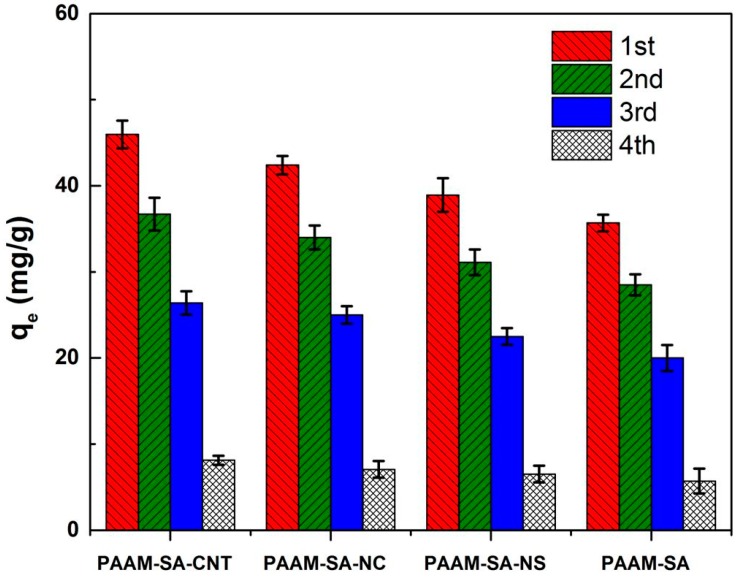
Four adsorption–desorption cycles of Cu^2+^ on the prepared hydrogels.

The results of the reusability of the four types of hydrogels are displayed in Figure 8. It can be seen that although there was a slight drop in the adsorption capacities of the hydrogels as the regeneration cycles increased, the hydrogels exhibited a satisfactory adsorption capacity over two to three adsorption–desorption cycles. This phenomenon demonstrated that the obtained hydrogels could be employed through multiple adsorption–desorption processes. Thus, the hybrid hydrogels have a great potential to serve as adsorbents for the removal of Cu^2+^ from wastewater.

## 4. Conclusions

In this work, the IPN-structured hydrogels were prepared by in situ polymerization of acrylamide (AAM) and sodium alginate (SA). The well-dispersed CNTs, NCs, and NSs were incorporated into the PAAM-SA matrix. The neat hydrogel with a water content of 85.4% achieved a compression strength of 0.27MPa at ε = 60% and a maximum adsorption capacity of 38.9 mg/g. After incorporating only 0.036 g nanofillers into a 30 g polymer matrix, the obtained hydrogels exhibited a higher compressive strength, a higher fatigue resistance, and a better adsorption capacity. The obtained PAAM-SA-NS exhibited the highest compression strength of 1.3 MPa at ε = 60%, which was 4.8 times higher than that of PAAM-S,; whereas PAAM-SA-CNT possessed the largest adsorption capacity owing to the multi-walled hollow nanostructure of CNT, and the adsorption capacity of PAAM-SA-CNT was 1.28-fold higher than that of PAAM-SA. The prepared hydrogels can be potential candidates for use as absorbents to treat wastewater.

## Figures and Tables

**Table 1:** Physical and compression properties of hydrogels.

Hydrogels	Stress σ at ε = 60% (MPa)	Energy Absorption at ε = 60% (kJ/m^3^)	Specific Stress σ_s_ at ε = 60% (MPa·cm^3^/g)
PAAM-SA	0.27 ± 0.1	3.3 ± 0.32	≈0.2
PAAM-SA-CNT	0.43 ± 0.17	5.4 ± 0.4	≈0.3
PAAM-SA-NC	0.73 ± 0.21	8.2 ± 0.38	≈0.5
PAAM-SA-NS	1.3 ± 0.23	13.5 ± 0.45	≈0.9

**Table 2:** Cu^2+^ adsorption capacities for various adsorbents.

Adsorbent	Isotherm	Temperature (°C)	*q_e_*(mg/g)	Reference
Konjac glucomannan-poly(acrylic acid) hydrogel	L	25	27	[44]
Poly(vinylpyrrolidone/acrylic acid) hydrogel	L	25	23	[45]
Fe_3_O_4_-SiO_2_ NPs	L	25	29.85	[19]
Poly(hydroxyethyl methacrylate/maleamic acid) hydrogel	L	25	10.24	[21]
Poly(acrylamide-co-sodium methacrylate) hydrogel	L	25	24.05	[46]
Chitosan	L	25	35.46	[47]
PAAM-SA-CNT	L	25	46	This work

**Table 3:** Comparison of Langmuir and Freundlich isotherm parameters with experimental adsorption data of Cu^2+^ on the hydrogels at 25 °C.

Sample	*q_m_* (exp) (mg/g)	Langmuir				Freundlich		
		*q_m_* (mg/g)	*K_L_* (L/mg)	*R* ^2^	*R_L_*	*K_F_* (mg/g)	*1*/*n*	*R* ^2^
PAAM-SA-CNT	46	48.5	0.11	0.998	0.06–0.31	13.27	0.274	0.934
PAAM-SA-NC	42.4	46.7	0.08	0.994	0.08–0.38	11.2	0.291	0.929
PAAM-SA-NS	38.9	42.5	0.09	0.997	0.07–0.35	11.68	0.264	0.918
PAAM-SA	35.7	40.8	0.06	0.993	0.1–0.45	8.11	0.318	0.933

## Supplementary Materials

The following are available online at https://www.mdpi.com/2073-4360/11/8/1239/s1.
