# Supplementary material for: Assembly of Polyacrylamide-Sodium Alginate-Based Organic-Inorganic Hydrogel with Mechanical and Adsorption Properties
Source: Polymers (Basel). 2019 Jul 26;11(8):1239. doi: 10.3390/polym11081239 (PMC6722572; doi:10.3390/polym11081239)
Supplement: Supplementary file 1 [file polymers-11-01239-s001.pdf]

1. Chemical structure of PAAM, SA, CNT, NC and NS

For better understanding the FTIR results, the spectra of PAAM, SA, CNT, NC and NS present in Figure S1. It can be seen that the absorption peaks at 3340 cm^{-1} which were attributed to the stretching of –OH was shown in all the spectra, suggesting the presence of hydroxyl groups on the surface of three inorganic nanoparticles. The IR spectrum of PAAM exhibited characteristic peaks at 3426 and 3228 cm^{-1}, representing asymmetric and symmetric N–H stretching vibrations [1]. The peaks at 1135 cm^{-1} implied the C–N and N–H at $CONH_2$ group [2] and the bands at 1662, 1610 and 2930 cm^{-1} assigned to CO stretching, NH bending, CH stretching, respectively. In the spectrum of SA, two bands at 1617 and 1413 cm^{-1} corresponded to COO- group and one sharp peak at 1035 cm^{-1} were responsible for C-O vibration. Moreover, the characteristic peak appeared at 813 cm^{-1} on SA depicted the Na-O vibration [3]. Due to the vibration of the carbon skeleton, the spectrum of CNT showed a characteristic band at 1560 cm^{-1} [4]. The absorption peaks at 1640 cm^{-1} and 1099 cm^{-1} may originate to O-H vibrations of adsorbed water and C-O-C bending on CNT [5]. For IR spectrum of NC, the absorption bands in the range of 838 cm^{-1}-450 cm^{-1} were attributed to –OH bending and M–O (M denotes Si or other metal cations existed in clay) [6]. Particularly, the two peaks at 796 and 592 cm^{-1} of NC were ascribed to the deformation vibration of Al–O–Si bonds [7]. The analyzing of NS spectrum was specified in the manuscript.

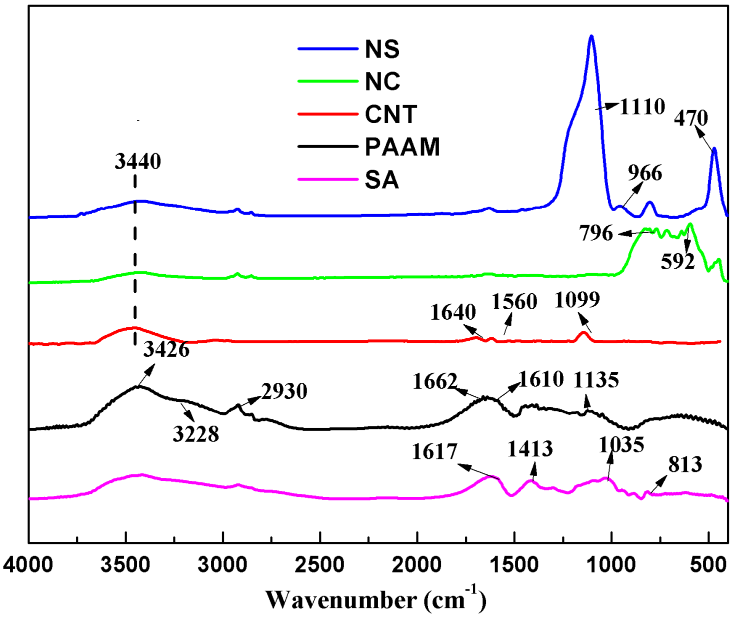

Figure S1. FTIR spectra of PAAM, SA, CNT, NC and NS.

2. The effects of pH and adsorbent dosage on adsorption

The adsorption experiments were carried out by using 120mg prepared hydrogels in 150 ml of Cu^{2+} solution at 25°C. For investigating the effect of pH on adsorption, the concentration of Cu^{2+} was selected as 150mg/L. For inquiring the effect of hydrogel dosages on adsorption, various Cu^{2+} concentrations (20-120mg/L) were applied to the experiments. After reaching absorbent equilibrium, the adsorbents were removed from the solution. The concentrations of Cu^{2+} were determined by Flame Atomic Absorption Spectrometer (FAAS, TAS-990, Persee,

Beijing, China).

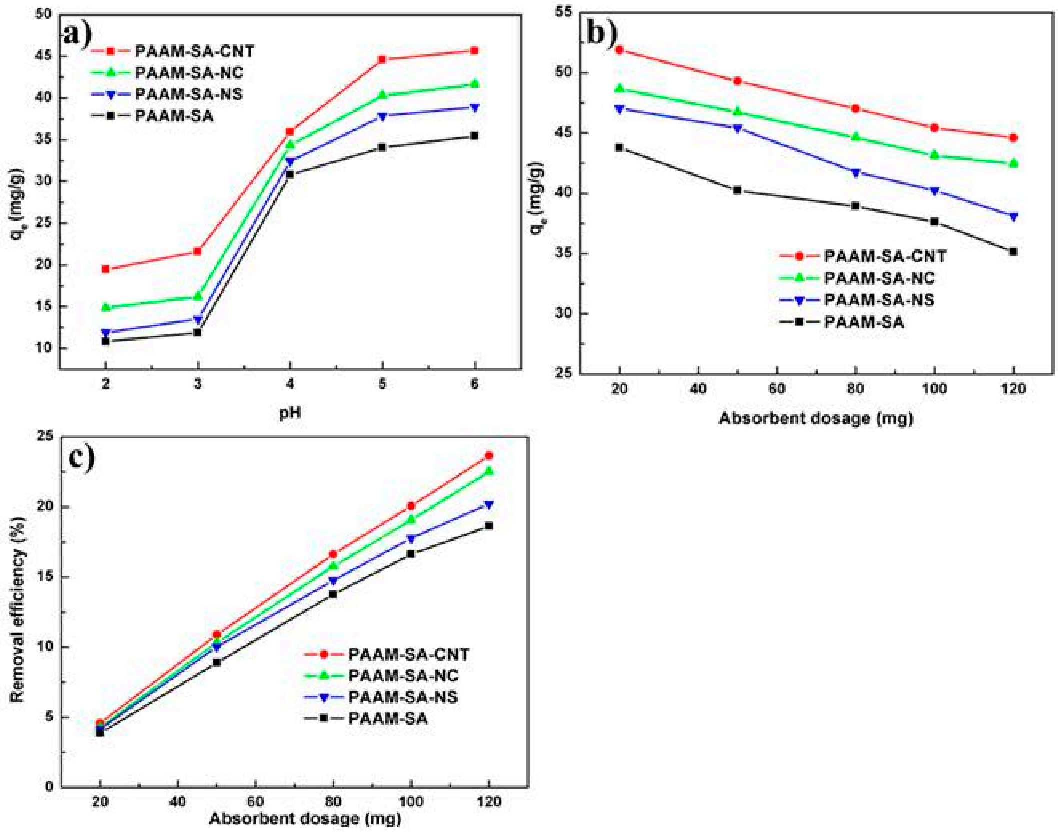

Figure S2. The adsorption of Cu^{2+} on hydrogels: (a) the effects of pH; (b) the absorbent dosages versus the adsorption capacities at equilibrium; (c) the absorbent dosages versus removal efficiencies.

Figure S2a displays the absorption capacities of the hydrogels to Cu^{2+} at pH ranging from 2 to 6. In this study, pH above 6.0 was not studied because the $Cu(OH)_2$ would be produced at pH higher than 6.0. At pH ranging of 2-6, the adsorption abilities of the hydrogels were gradually increased with the increasing of pH. The adsorption rates were low at pH level of 2-3. The possible reason was that the -NH_2 groups in the PAAM chains were protonated resulting in a positively surface charge of the hydrogels. Hence, the electrostatic repulsion between positively charged hydrogel surface and positively charged Cu^{2+} inhibited the adsorption of Cu^{2+} on hydrogels. In addition, there were more H^+ at lower pH levels and these H^+ could compete with Cu^{2+} for the adsorption site, which also led to a decrease in adsorption capacity [8]. With the increasing of pH values, -NH_2 groups were not ionized whereas -COOH and –OH groups were ionized. The negatively charged –COO^- and -O^- groups improved the adsorption by electrostatic interactions with Cu^{2+} [9]. The obtained results are in accordance with previous literatures [8-10]. The effects of the amounts of adsorbent on the adsorption of Cu^{2+} are showed in Figure S2b and S2c. The adsorption capacities decreased with the increasing of the sorbent dosage from 20 to 120mg, however, the total removal efficiencies of Cu^{2+} gradually increased. This phenomenon was attributed to the number of active groups on the surface of the hydrogel increased for adsorbing Cu^{2+} [10, 11].

References

1. Gad, Y.H.; Aly, R.O., and Abdel-Aal, S.E., Synthesis and characterization of Na-alginate/acrylamide hydrogel and its application in dye removal.*Journal of Applied Polymer Science*, **2011**, *120*, 1899–1906.
2. Al-Kahtani, A.A. and Sherigara, B.S., Semi-interpenetrating network of acrylamide-grafted-sodium alginate microspheres for controlled release of diclofenac sodium, preparation and characterization. *Colloids and Surfaces B-Biointerfaces*, **2014**, *115*, 132–138.
3. Li, P.; Yan, L.; Huang, J.; Li, J.; Gong, J., and Ma, J., Microfluidic Fabrication of Highly Stretchable and Fast Electro-responsive Graphene Oxide/Polyacrylamide/Alginate Hydrogel Fibers. *European Polymer Journal*, **2018**, *103*, 335–341.
4. Poernomo, G.; Cong, G.; Xianghua, S.; Quanyuan, Z.; Leong, S.S.J.; Chuyang, T.; Yuan, C.; Chan-Park, M.B.; Matthew Wook, C. and Kean, W., Hollow fiber membrane decorated with Ag/MWNTs: toward effective water disinfection and biofouling control. *ACS Nano*, **2011**, *5*, 10033–10040.
5. Han, J.; Wang, H.; Yue, Y.; Mei, C.; Chen, J.; Huang, C.; Wu, Q. and Xu, X., A self-healable and highly flexible supercapacitor integrated by dynamically cross-linked electro-conductive hydrogels based on nanocellulose-templated carbon nanotubes embedded in a viscoelastic polymer network. *Carbon*, **2019**, *149*, 1–18.
6. Zhang, J. and Wang, A., Study on superabsorbent composites. IX: Synthesis, characterization and swelling behaviors of polyacrylamide/clay composites based on various clays. *Reactive and Functional Polymers*, **2007**, *67*, 737–745.
7. Mao, X.; Wang, L.; Gu, S.; Duan, Y.; Zhu, Y.; Wang, C., and Lichtfouse, E., Synthesis of a three-dimensional network sodium alginate-poly(acrylic acid)/attapulgite hydrogel with good mechanic property and reusability for efficient adsorption of Cu^{2+} and Pb^{2+}. *Environmental Chemistry Letters*, **2018**, 1–6.
8. Tong, D.; Fang, K.; Yang, H.; Wang, J.; Zhou, C., and Yu, W., Efficient removal of copper ions using a hydrogel bead triggered by the cationic hectorite clay and anionic sodium alginate. *Environmental Science and Pollution Research*, **2019**, *26*, 16482–16492.
9. Wang, M.; Li, X.; Zhang, T.; Deng, L.; Li, P.; Wang, X., and Hsiao, B.S., Eco-friendly poly(acrylic acid)-sodium alginate nanofibrous hydrogel: A multifunctional platform for superior removal of Cu(II) and sustainable catalytic applications. *Colloids and Surfaces A-Physicochemical and Engineering Aspects*, **2018**, *558*, 228–241.
10. Milosavljevic, N.; Debeljkovic, A.; Krusic, M.K.; Milasinovic, N.; Uzum, O.B., and Karadag, E., Application of Poly(acrlymide-co-sodium methacrylate) Hydrogels in Copper and Cadmium Removal from Aqueous Solution. *Environmental Progress and Sustainable Energy*, **2014**, *33*, 824–834.
11. Zou, X.; Zhang, H.; Chen, T.; Li, H.; Meng, C.; Xia, Y., and Guo, J., Preparation and characterization of polyacrylamide/sodium alginate microspheres and its adsorption of MB dye. *Colloids and Surfaces A: Physicochemical and Engineering Aspects*, **2019**, *567*, 184–192.
